# Preparation and Characterization of Magnetic Metal–Organic Frameworks Functionalized by Ionic Liquid as Supports for Immobilization of Pancreatic Lipase

**DOI:** 10.3390/molecules27206800

**Published:** 2022-10-11

**Authors:** Moju Li, Xusheng Dai, Aifeng Li, Qi Qi, Wenhui Wang, Jia Cao, Zhenting Jiang, Renmin Liu, Hongbo Suo, Lili Xu

**Affiliations:** 1 School of Chemistry and Chemical Engineering, Liaocheng University, Liaocheng 252059, China; 2 School of Pharmaceutical Sciences, Liaocheng University, Liaocheng 252059, China

**Keywords:** metal–organic framework, magnetic nanoparticles, lipase, ionic liquids, immobilization

## Abstract

Enzymes are difficult to recycle, which limits their large-scale industrial applications. In this work, an ionic liquid-modified magnetic metal–organic framework composite, IL-Fe_3_O_4_@UiO-66-NH_2_, was prepared and used as a support for enzyme immobilization. The properties of the support were characterized with X-ray powder diffraction (XRD), Fourier-transform infrared (FTIR) spectra, transmission electron microscopy (TEM), scanning electronic microscopy (SEM), and so on. The catalytic performance of the immobilized enzyme was also investigated in the hydrolysis reaction of glyceryl triacetate. Compared with soluble porcine pancreatic lipase (PPL), immobilized lipase (PPL-IL-Fe_3_O_4_@UiO-66-NH_2_) had greater catalytic activity under reaction conditions. It also showed better thermal stability and anti-denaturant properties. The specific activity of PPL-IL-Fe_3_O_4_@UiO-66-NH_2_ was 2.3 times higher than that of soluble PPL. After 10 repeated catalytic cycles, the residual activity of PPL-IL-Fe_3_O_4_@UiO-66-NH_2_ reached 74.4%, which was higher than that of PPL-Fe_3_O_4_@UiO-66-NH_2_ (62.3%). In addition, kinetic parameter tests revealed that PPL-IL-Fe_3_O_4_@UiO-66-NH_2_ had a stronger affinity to the substrate and, thus, exhibited higher catalytic efficiency. The results demonstrated that Fe_3_O_4_@UiO-66-NH_2_ modified by ionic liquids has great potential for immobilized enzymes.

## 1. Introduction

As renewable green biocatalysts, enzymes require mild reaction conditions and have strong substrate specificity, high catalytic efficiency, and easily adjustable catalytic activity, and produce no environmental pollution [1,2,3]. They have been widely used in many fields, such as food processing, biopharmaceuticals, the energy and chemical industries, and environmental catalysis [4,5,6]. However, soluble enzymes are susceptible to the reaction environment and have poor stability at extreme pH values, high temperatures, and in some organic solvents. The recovery of soluble enzymes is complex, the recycling rate is low, and they are relatively expensive [1,7], which all limit the large-scale industrial utilization of soluble enzymes [8]. Immobilizing enzymes provide broad prospects for efficiently utilizing enzymes.

Immobilized enzyme technologies use physical or chemical methods to combine an enzyme with a support to improve the catalytic activity and operational stability of the enzyme [5,7]. The properties of immobilized enzymes are largely influenced by the immobilization support and immobilization method [9,10]. Many recent reports have discussed the preparation of simple, stable, and efficient support materials, including organic polymers, carbon nanotubes, mesoporous silica, magnetic nanoparticles, and metal–organic frameworks [11,12,13,14,15].

Metal–organic frameworks (MOFs) have tunable structures, abundant coordination sites, open framework structures, and diverse pore sizes, all of which can create a stable microenvironment for enzyme molecules, limit the leakage of enzyme molecules, and significantly improve the enzyme loading and substrate transfer efficiency. MOFs can maintain enzyme activity, even under extreme conditions [15,16,17]. Although MOFs have obvious structural advantages, there are some problems related to instability that have attracted attention, such as weak mechanical strength and difficult recovery. Researchers have proposed methods to combine MOFs with functional materials to improve the stability of MOFs. Magnetic MOF composites are some of the functionalized MOFs that have been reported, combining the advantages of magnetic nanoparticles (MNPs) and those of MOFs, and they show excellent application value in immobilized enzymes [18,19]. Zhong et al. used a simple one-pot method to synthesize a novel magnetic MOF with a large specific surface area and high enzyme-carrying capacity. The activity of immobilized trypsin was 2.6 times higher than that of the soluble enzyme, and the composite could be easily magnetically recovered [20].

Ionic liquids (ILs) are salts prepared from organic cations and organic or inorganic anions that take on a liquid form at or around room temperature [21]. ILs are not volatile, have good thermal stability, are chemically stable, can reduce the generation of harmful organic wastes, and are used as green solvents in enzyme-catalyzed reactions [22]. Selecting different anions and cations can adjust the polarity to improve the microenvironment of enzymes [23]. More recently, ILs have been used in immobilized enzymes. Using the ionic liquid to modify the support can promote the catalytic activity of the enzyme and also improve the utilization efficiency of the ionic liquid compared with using it as a solvent [21,24,25]. Barbosa et al. reported that IL [P_666(14)_][NTf_2_] composed of cations with longer alkyl side chains and more hydrophobic anions exhibited the best effect on BCL. When IL [P_666(14)_][NTf_2_] was applied to immobilize the enzyme, the immobilization efficiency and activity increased and BCL retained more than 50% of its initial activity after 26 repeated uses [26].

**Scheme 1:**
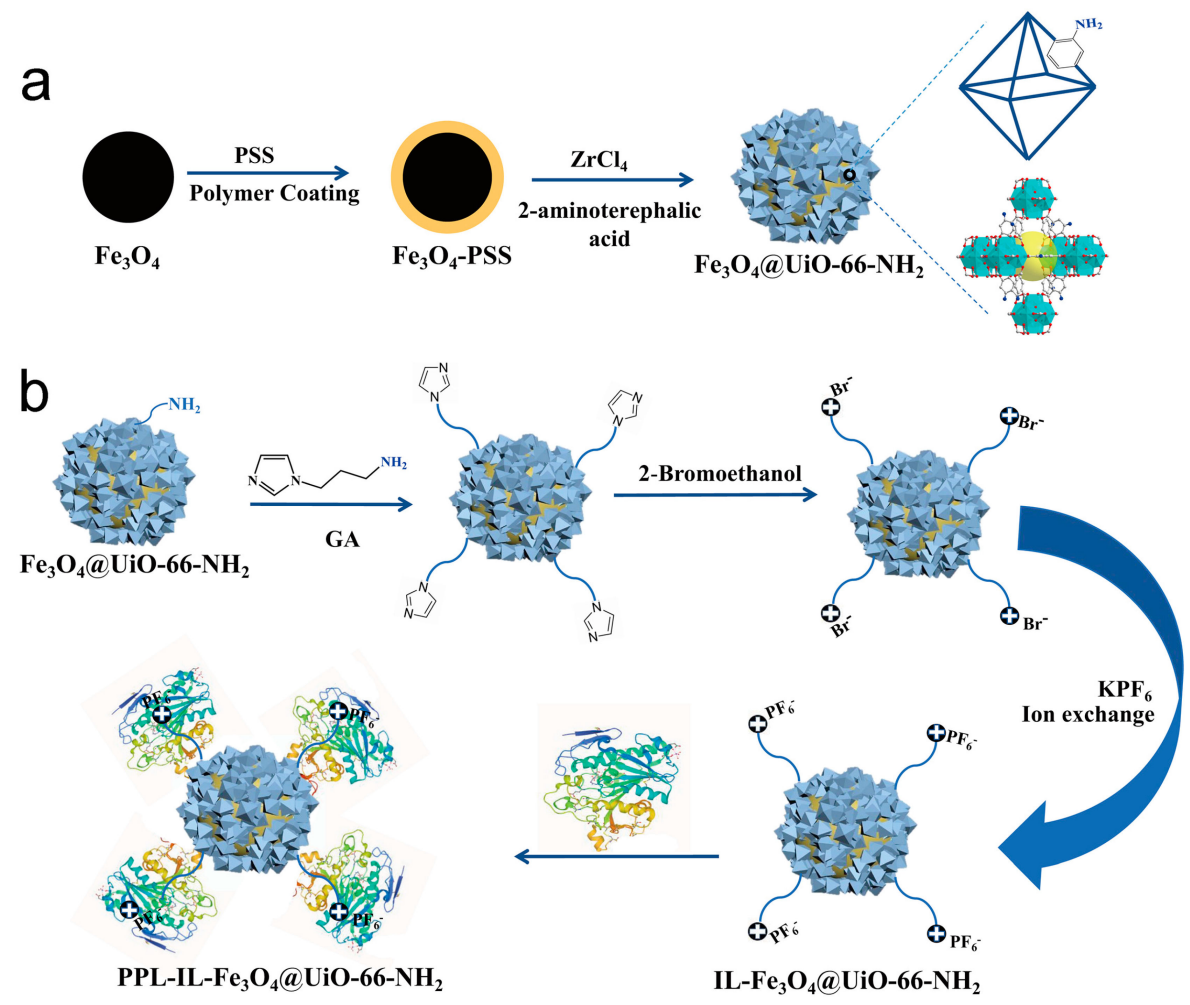
(**a**) Schematic illustration for the synthesis of core–shell magnetic MOF composite Fe_3_O_4_@UiO-66-NH_2_; (**b**) schematic diagram of IL modification of Fe_3_O_4_@UiO-66-NH_2_ and immobilization of PPL.

In this work, a Fe_3_O_4_@UiO-66-NH_2_ composite with a core–shell structure was modified with imidazole-based ionic liquids and then used as a support to immobilize lipase through an adsorption method, and the preparation process is shown in Scheme 1. IL-Fe_3_O_4_@UiO-66-NH_2_ provided an appropriate microenvironment for the immobilized enzyme and showed an excellent magnetic response. Therefore, the catalytic activity, stability, reusability, and kinetic parameters of the immobilized enzyme were researched. This work is the first application of ionic liquid-modified Fe_3_O_4_@UiO-66-NH_2_ in the field of immobilized lipases and provides a reference for applying magnetic MOF composites for immobilized enzymes.

## 2. Results and Discussion

### 2.1. Preparation and Characterization of Supports

**Figure 1:**
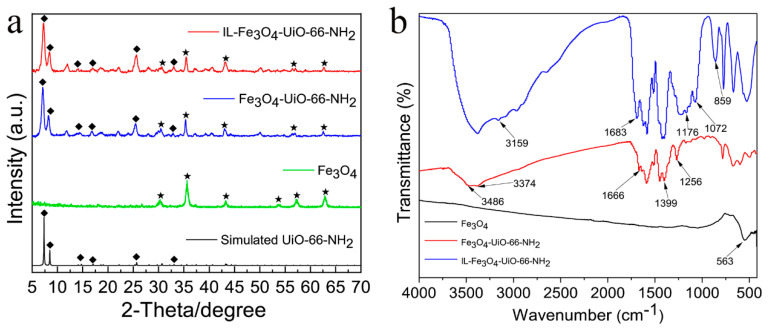
(**a**) XRD of simulated UiO-66-NH_2_ (green), Fe_3_O_4_ (black), Fe_3_O_4_@UiO-66-NH_2_ (blue), IL-Fe_3_O_4_@UiO-66-NH_2_ (red), (◆ indicates the diffraction peak of UiO-66-NH_2_, ★ indicates the diffraction peak of Fe_3_O_4_); (**b**) FTIR spectra of Fe_3_O_4_ (black), Fe_3_O_4_@UiO-66-NH_2_ (red), and IL-Fe_3_O_4_@UiO-66-NH_2_ (blue).

Figure 1a shows the XRD patterns of Fe_3_O_4_, simulated UiO-66-NH_2_, Fe_3_O_4_@UiO-66-NH_2_, and IL-Fe_3_O_4_@UiO-66-NH_2_. For Fe_3_O_4_ (green), the pattern exhibited intense reflections at 2θ = 30.18°, 35.54°, 43.39°, 53.57°, 57.31°, and 62.94°, corresponding to the (2 2 0), (3 1 1), (4 0 0), (4 2 2), (5 1 1), and (4 4 0) crystal planes, of the crystal structure of Fe_3_O_4_ (JCPDS 19-0629) [27]. The Fe_3_O_4_@UiO-66-NH_2_ pattern had additional strong reflections at 2θ = 7.06°, 8.19°, 14.37°,16.94°, 24.93°, and 32.78°, corresponding to the (1 1 1), (2 0 0), (2 2 2), (4 0 0), (6 0 0), and (6 4 0) planes of UiO-66-NH_2_ [28]. In addition, the pattern of IL-Fe_3_O_4_@UiO-66-NH_2_ (red) retained the characteristic peaks of both Fe_3_O_4_ and UiO-66-NH_2_, demonstrating that the crystal structure of Fe_3_O_4_@UiO-66-NH_2_ was not destroyed during the ionic liquid modification.

The chemical composition of Fe_3_O_4_, Fe_3_O_4_@UiO-66-NH_2_, and IL-Fe_3_O_4_@UiO-66-NH_2_ was investigated using FTIR spectroscopy (Figure 1b). The vibrational absorption peak of Fe-O in the Fe_3_O_4_ nanoparticles’ spectrum appeared at 563 cm^−1^ [29]. For the spectrum of Fe_3_O_4_@UiO-66-NH_2_ (red), the characteristic absorption peaks at 1256 cm^−1^ and 1399 cm^−1^ were attributed to the typical C-N stretching vibration of aromatic amines. The N-H bending vibration exhibited an intense band at 1666 cm^−1^. The characteristic absorption peaks at 3374 cm^−1^ and 3486 cm^−1^ were assigned to the asymmetric and symmetric N-H stretching of the MOF’s structure, indicating that UiO-66-NH_2_ was successfully coated on Fe_3_O_4_ nanoparticles [30]. For the spectra of IL-Fe_3_O_4_@UiO-66-NH_2_ (blue), the characteristic peak at 1683 cm^−1^ indicated the generation of C=N imine bonds due to the introduction of API, which underwent a Schiff base reaction. The peak near 1176 cm^−1^ was the stretching vibration of the imidazole ring, and the absorption peak at 3159 cm^−1^ represents the C-H stretching vibration of the imidazole ring. The characteristic absorption peak at 1072 cm^−1^ arose from the C-N stretching of primary amines [31]. The peak around 859 cm^−1^ was identified as the characteristic absorption of P-F in the anion [PF_6_]^−^ of IL [32]. The results indicate that the MOF composites were modified successfully by the IL.

**Figure 2:**
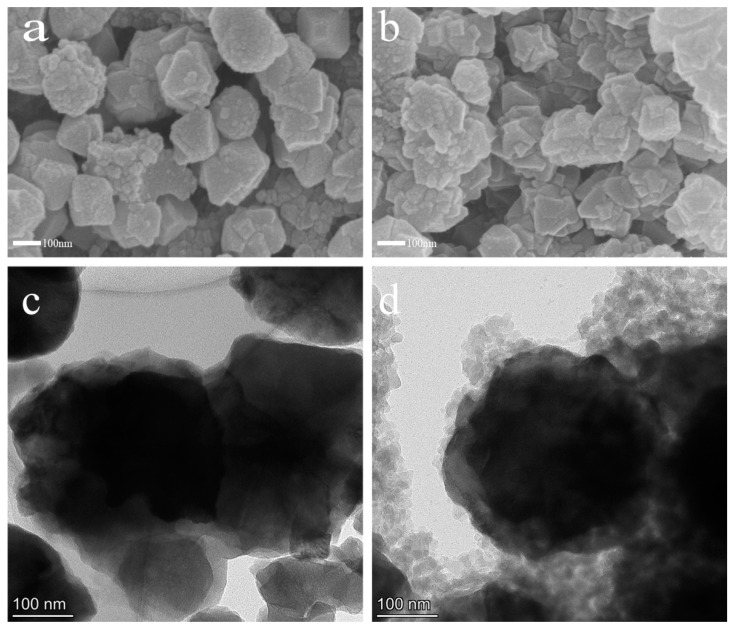
SEM images of Fe_3_O_4_@UiO-66-NH_2_ (**a**) and IL-Fe_3_O_4_@UiO-66-NH_2_ (**b**); TEM images of Fe_3_O_4_@UiO-66-NH_2_ (**c**) and IL-Fe_3_O_4_@UiO-66-NH_2_ (**d**).

**Figure 3:**
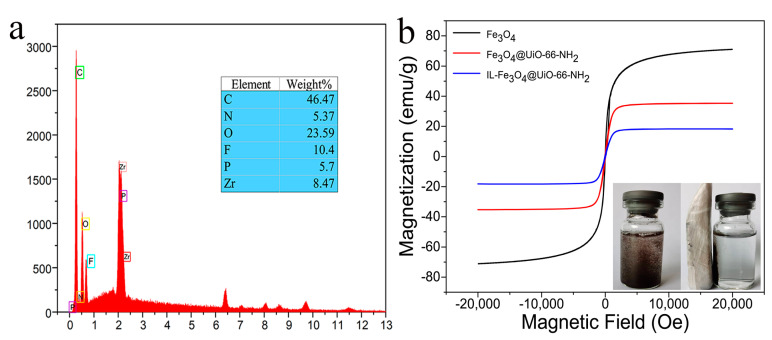
(**a**) EDS analysis of IL-Fe_3_O_4_@UiO-66-NH_2_; (**b**) the hysteresis loops of the supports; the insert is a picture of the magnetic response.

The morphologies and microstructures of the supports were investigated by SEM and TEM. As shown in the SEM image in Figure 2a,b, Fe_3_O_4_@UiO-66-NH_2_ presented an octahedral shape [33]. The TEM images (Figure 2c,d) showed that the average diameter of Fe_3_O_4_@UiO-66-NH_2_ was in the range of 280–300 nm and indicated a distinct core–shell structure [28]. The average diameter of IL-Fe_3_O_4_@UiO-66-NH_2_ is also in the range of 280–300 nm, which indicates that the ionic liquid modification process did not significantly change the particle size. The elemental and compositional analysis of IL-Fe_3_O_4_@UiO-66-NH_2_ showed that the P and F content was 5.7% and 10.1%, respectively (Figure 3a). This proved that the anion of IL was [PF_6_]^−^, further demonstrating the successful modification of Fe_3_O_4_@UiO-66-NH_2_ by this ionic liquid.

The magnetic hysteresis loops of Fe_3_O_4_, Fe_3_O_4_@UiO-66-NH_2_, and IL-Fe_3_O_4_@UiO-66-NH_2_ are shown in Figure 3b. The saturation magnetization values of Fe_3_O_4_, Fe_3_O_4_@UiO-66-NH_2_, and IL-Fe_3_O_4_@UiO-66-NH_2_ were 71.02 emu/g, 35.31 emu/g, and 18.25 emu/g, respectively, showing that the magnetic intensity decreased after forming Fe_3_O_4_@UiO-66-NH_2_. This was because the Fe_3_O_4_ nanoparticles were coated with a core–shell structure, which decreased the magnetization of the supports [34]. Although the magnetic intensity was further reduced after ionic liquid modification, magnetic separation could be easily performed using an external magnetic field, as shown in the illustration in the inset of Figure 3b.

**Figure 4:**
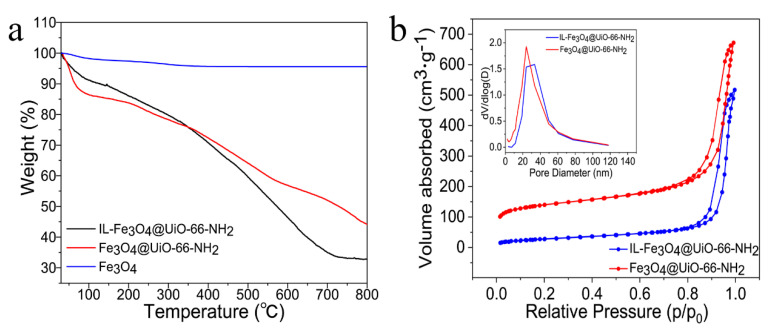
(**a**) TGA curves of the supports; (**b**) N_2_ adsorption-desorption isotherms of Fe_3_O_4_@UiO-66-NH_2_ and IL-Fe_3_O_4_@UiO-66-NH_2_ and pore size distribution curves of Fe_3_O_4_@UiO-66-NH_2_ and IL-Fe_3_O_4_@UiO-66-NH_2_.

The TGA curves shown in Figure 4a were used to determine the thermal behaviors of Fe_3_O_4_, Fe_3_O_4_@UiO-66-NH_2_, and IL-Fe_3_O_4_@UiO-66-NH_2_. The weight loss occurring before 150 °C was derived from solvent evaporation from the sample. According to the curve of Fe_3_O_4_@UiO-66-NH_2_, the weight loss in the temperature range of 150–350 °C was attributed to dehydroxylation of the carboxylate ligand in UiO-66-NH_2_. The 30.7% weight loss from 350 to 800 °C was due to the combustion of the NH_2_-BDC connector and the decomposition of the framework [35,36]. The curve of IL-Fe_3_O_4_@UiO-66-NH_2_ decreased by 42.5% in the temperature range of 350–800 °C due to the decomposition of the imidazole-based ionic liquid from 350 °C [32]. The TGA results show that UiO-66-NH_2_ formed outside Fe_3_O_4_, and the ILs were grafted onto the Fe_3_O_4_@UiO-66-NH_2_ surface.

The porous structure of Fe_3_O_4_@UiO-66-NH_2_ and IL-Fe_3_O_4_@UiO-66-NH_2_ was studied via nitrogen adsorption–desorption isotherms. The curve in Figure 4b shows a mixture of type I and IV adsorption and desorption, indicating that both micropores and mesopores were present in the sample [28]. The BET surface area of Fe_3_O_4_@UiO-66-NH_2_ was 494.73 m^2^/g, while that of IL-Fe_3_O_4_@UiO-66-NH_2_ decreased to 102.84 m^2^/g. The reduction in the BET surface area mainly occurred due to the occupation of pores by the introduced ionic liquids. ILs enhance the enzyme–support interactions through ionic interactions, π–π stacking, hydrophobic interactions, etc. Hence, despite the lower BET surface area, the loading capacity increased (Table 1). This also indicated that lipase was immobilized on the surface and did not enter the pores of the MOFs.

### 2.2. Results of Immobilized Lipase and Activity Test

The immobilization results are shown in Table 1. The enzyme loading of Fe_3_O_4_@UiO-66-NH_2_ was 153.8 mg/g, and the specific activity of PPL-Fe_3_O_4_@Ui_O_-66-NH_2_ was 1.9 U/mg, which was higher than that of soluble PPL (0.9 U/mg). The loading capacity of PPL-IL-Fe_3_O_4_@UiO-66-NH_2_ increased to 193.5 mg/g, and the specific activity reached 2.03 U/mg, which was approximately 2.3 times higher than that of soluble PPL. Fe_3_O_4_@UiO-66-NH_2_ had good biocompatibility, while its MOF backbone provided rigid protection for lipase, helping it to maintain the structure and improve the activity of lipase. Modification by the ionic liquid provided a more suitable microenvironment for lipase, and lipase could also interact with the imidazole-based IL through π–π stacking. The introduction of IL helped to stabilize the open-cap conformation of the enzyme by interfacial activation, which facilitated interactions between the active site and substrate and increased the stability. Thus, PPL-IL-Fe_3_O_4_@UiO-66-NH_2_ exhibited higher activity [2,37].

### 2.3. Effects of pH and Temperature on Lipase Activity

**Figure 5:**
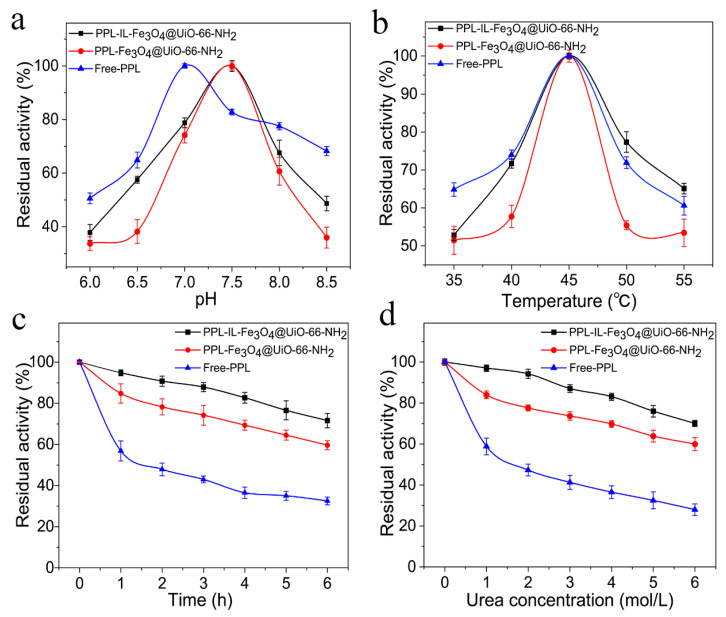
The effects of (**a**) pH and (**b**) temperature on the activity of free or immobilized lipase; (**c**) thermal stability of soluble and immobilized PPL; (**d**) denaturant tolerance of soluble and immobilized PPL.

At a certain temperature, the optimal reaction pH of soluble PPL, PPL-Fe_3_O_4_@UiO-66-NH_2_, and PPL-IL-Fe_3_O_4_@UiO-66-NH_2_ was screened. The activity at the optimum pH was defined as 100%, and the residual activity at different pH levels was calculated, as shown in Figure 5a. The results showed that the optimal reaction pH for soluble PPL was 7.0, but it was 7.5 for PPL-Fe_3_O_4_@UiO-66-NH_2_ and PPL-IL-Fe_3_O_4_@UiO-66-NH_2_. This was probably because the effects of H^+^ on the immobilized enzyme conformation were reduced in a weakly alkaline environment, which gave the immobilized enzyme higher activity at high pH values [25,37]. Similar to the screening method to determine the optimal reaction pH, the optimal reaction temperatures for soluble PPL, PPL-Fe_3_O_4_@UiO-66-NH_2_, and PPL-IL-Fe_3_O_4_@UiO-66-NH_2_ were screened. The results in Figure 5b show that the optimal reaction temperature for both soluble and immobilized PPL was 45 °C. In addition, PPL-IL-Fe_3_O_4_@UiO-66-NH_2_ exhibited the highest residual activity in the temperature range of 45–55 °C. The magnetic MOF composite support and IL modification helped to maintain the conformation of the immobilized enzyme and provided greater temperature tolerance [38].

### 2.4. Thermal Stability Study

The thermal stability of immobilized enzymes is a key issue to consider in catalytic reactions [39]. The enzyme activity measured at 0 h was defined as 100% residual activity. As shown in Figure 5c, the activities of soluble PPL, PPL-Fe_3_O_4_@UiO-66-NH_2_, and PPL-IL-Fe_3_O_4_@UiO-66-NH_2_ decreased with the incubation time. After 6 h of incubation, the residual activity of PPL-Fe_3_O_4_@UiO-66-NH_2_ and PPL-IL-Fe_3_O_4_@UiO-66-NH_2_ was 59.6% and 71.6%, respectively. However, only 33.9% of the residual activity of soluble PPL was retained. Compared with soluble PPL, the rigid MOF structure protected lipase by confining the enzyme within a biocompatible microenvironment that prevented its leakage. The imidazole-based IL is also beneficial for preserving the structural integrity of lipase, which can limit enzyme inactivation [37,40].

### 2.5. Research on Anti-Denaturant Properties

The inherent fragility of enzymes renders them unstable in harsh environments, such as in the presence of denaturants, leading to activity loss and shorter lifetimes [41]. Therefore, the anti-denaturant properties of the enzyme were evaluated by incubating it in a urea solution, and the results are shown in Figure 5d. The activity of both soluble and immobilized lipase decreased upon increasing the concentration of urea. The soluble PPL retained only 26.9% of its activity after incubation in 6 M urea solution. However, PPL-Fe_3_O_4_@UiO-66-NH_2_ and PPL-IL-Fe_3_O_4_@UiO-66-NH_2_ retained 60.6% and 70.3% of their activity, respectively. In this process, urea denatured lipase by breaking the hydrogen bonds in its structure and preferentially interacting with its lipase surface. The IL-Fe_3_O_4_@UiO-66-NH_2_ support protected the structure of the enzyme and reduced the contact between the active center and denaturant [42]. Moreover, the introduction of the ionic liquid further enhanced the tolerance of the enzyme to the denaturant due to the strong hydrogen bonding of [PF_6_]^−^, which attenuated the denaturing effect of urea and protected the lipase from induced inactivation [25].

### 2.6. Reusability Studies

**Figure 6:**
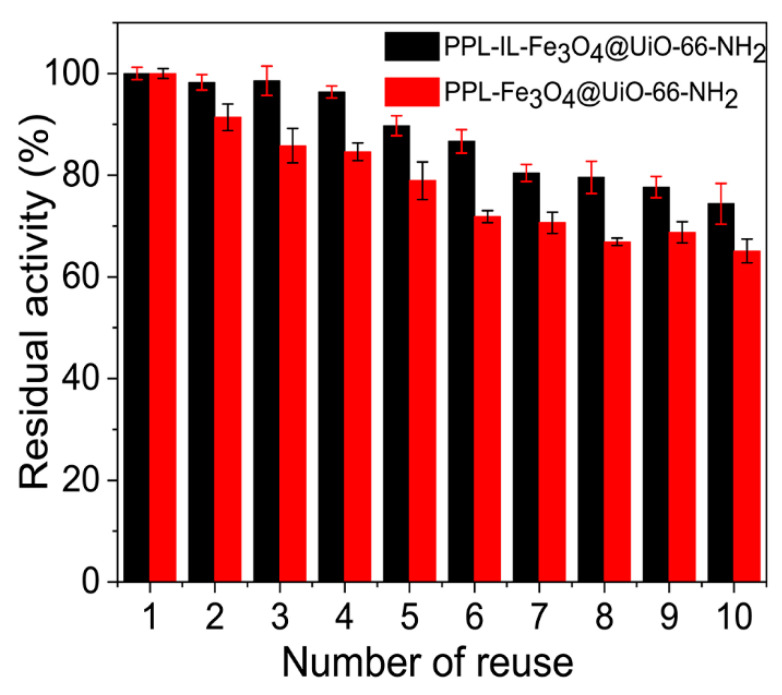
Reusability of the immobilized PPL for 10 cycles.

The reusability of immobilized enzymes is a key factor in making a process economically viable [43]. After each reaction was completed, the immobilized enzyme was magnetically separated, washed with PBS buffer solution (pH = 7.0), and added to a new reaction solution to conduct the next cycle. The enzyme activity measured in the first reaction was defined as the initial activity. The ratio of the activity during subsequent cycles to the initial activity was calculated as the relative activity. After repeated use for 10 consecutive cycles, the residual activity of PPL-Fe_3_O_4_@UiO-66-NH_2_ was still 62.3% (Figure 6), while that of PPL-IL-Fe_3_O_4_@UiO-66-NH_2_ was 74.4%. The MOF structure and IL modification reduced the leaching of the enzyme from the reaction mixture during multiple cycles. In cycling assays, the reduced residual activity may be a dual effect of mechanical damage and enzymatic inactivation. The magnetic properties of the immobilized enzyme enabled the convenient, rapid, and efficient separation from reaction mixtures using external magnets, enabling repeated use and reducing overall costs [44].

### 2.7. Kinetic Parameters

By measuring the initial reaction rate of the enzyme under different substrate concentrations, the Michaelis constant (*K*_m_) and maximum reaction rate (*V*_*max*_) were obtained by the double-reciprocal plot method (Table 2). The *K*_m_ values of PPL-Fe_3_O_4_@UiO-66-NH_2_ and PPL-IL-Fe_3_O_4_@UiO-66-NH_2_ were 75.48 and 71.74, respectively. The *K*_m_ value of PPL-IL-Fe_3_O_4_@UiO-66-NH_2_ was slightly lower, indicating that PPL-IL-Fe_3_O_4_@UiO-66-NH_2_ had a stronger affinity to the substrate [45]. The *V*_*max*_ of PPL-IL-Fe_3_O_4_@UiO-66-NH_2_ was higher than that of PPL-Fe_3_O_4_@UiO-66-NH_2_, showing that the substrate had a strong affinity with PPL-IL-Fe_3_O_4_@UiO-66-NH_2_ and high catalytic efficiency. This phenomenon may be due to a conformational change in the enzyme due to the introduction of IL, which made the interactions between the active site of the immobilized enzyme with the substrate more efficient, thereby increasing the catalytic efficiency [46,47].

## 3. Materials and Methods

### 3.1. Materials

Anhydrous ferric chloride (FeCl_3_), anhydrous sodium citrate, and anhydrous sodium acetate were obtained from Shanghai Macklin Biochemical Co., Ltd. (Shanghai, China). Meanwhile, 2-aminoterephalic acid, 2-bromoethanol, glutaraldehyde, and glyceryl triacetate were provided by Energy Chemical (Shanghai, China). Poly(sodium 4-styrene sulfonate) (PSS) was ordered from Shanghai Yien Chemical Technology Co., Ltd. (Shanghai, China). Zirconium chloride (ZrCl_4_), N-(3-Aminopropyl)-imidazole (API), and urea were purchased from Shanghai Aladdin Biochemical Technology Co., Ltd. (Shanghai, China). Porcine pancreatic lipase (PPL) was supplied by Sigma-Aldrich (Saint Louis, MO, USA). All the remaining reagents came from Shanghai Titan Scientific Co., Ltd. (Shanghai, China). Unless otherwise specified, all reagents used in this study were of analytical grade and used without further purification.

### 3.2. Preparation of Fe_3_O_4_ Nanoparticles

According to a previously reported protocol, Fe_3_O_4_ nanoparticles were prepared by the solvothermal method, with a minor modification [27]. FeCl_3_ (3.25 g, 4.0 mmol) and anhydrous sodium citrate (1.01 g, 0.78 mmol) were dissolved in 50 mL ethanol in a 100 mL beaker. Then, 1.2 g of anhydrous sodium acetate was added to the beaker and vigorously stirred for 30 min to form a homogeneous yellow solution. The mixed solution was transferred to a 100 mL PTFE-lined stainless steel autoclave and heated at 200 °C for 10 h. Then, the autoclave was allowed to naturally cool down. After this, the obtained black solid was washed several times with ethanol and deionized water and then vacuum-dried at 60 °C to obtain Fe_3_O_4_ nanoparticles.

### 3.3. Preparation of PSS-Fe_3_O_4_

The prepared Fe_3_O_4_ nanoparticles (1.0 g) were added to an aqueous solution of PSS (*w*/*v* = 0.3%, 400 mL), and the mixture was stirred at room temperature for 24 h to allow full contact. At the end of the reaction, PSS-Fe_3_O_4_ was collected with an external magnetic field, washed three times with deionized water, and dried in a vacuum oven at 60 °C.

### 3.4. Synthesis of Magnetic UiO-66-NH_2_ Composites

The Fe_3_O_4_@UiO-66-NH_2_ composites were synthesized by modifying a previously reported method [28]. PSS-Fe_3_O_4_ (1.3 g) was placed in 160 mL N,N-dimethylformamide (DMF), and then 1.0 g ZrCl_4_ and 0.75 g 2-aminoterephthalic acid were added sequentially. The mixed solution was transferred to a round-bottom flask, heated to reflux, and mildly stirred in an oil bath at 120 °C. The reaction was performed at this temperature for 4 h. Then, the product was collected from the mixed solution by an external magnetic field, washed with DMF three times, and redispersed into fresh solutions of ZrCl_4_ and 2-aminoterephthalic acid. After two cycles, Fe_3_O_4_@UiO-66-NH_2_ was washed several times alternately with ethanol and deionized water and then dried in a vacuum oven at 120 °C.

### 3.5. Preparation of IL-Modified Fe_3_O_4_@UiO-66-NH_2_

Fe_3_O_4_@UiO-66-NH_2_ (1.0 g) was dispersed in 150 mL of deionized water, and an appropriate amount of 50% glutaraldehyde was dropped into the solution. After continuous stirring at room temperature for 6 h, 3.0 mL of API was added and stirred for another 12 h. Next, the obtained solid was separated using a permanent magnet, rinsed several times with ethanol and acetonitrile, and dried for use in the next step. The solid was called API-Fe_3_O_4_@UiO-66-NH_2_. To synthesize IL on Fe_3_O_4_@UiO-66-NH_2_, API-Fe_3_O_4_@UiO-66-NH_2_ was mixed with 3.5 mL of bromoethanol in 100 mL of acetonitrile, and the mixture was heated at reflux at 82 °C for 12 h. Thereafter, solids were collected from the mixture and dispersed in an aqueous solution of KPF_6_ for ion exchange for 24 h. Finally, IL-Fe_3_O_4_@UiO-66-NH_2_ was separated using an external magnetic field, washed several times with deionized water, and dried in a vacuum oven at 120 °C.

### 3.6. Immobilization of Lipase

Lipase (1.5 g) was dissolved in 50 mL of phosphate-buffered saline (PBS; 0.025 M NaH_2_PO_4_ and Na_2_HPO_4_) at pH 7.0. Then, 0.3 g of the support was added and the mixture was placed in a constant-temperature shaker at 150 rpm and 35 °C for 3 h. The immobilized lipase was magnetically separated and washed several times with PBS (pH 7.0). The prepared immobilized lipase was freeze-dried and stored at 4 °C. The lipase protein content in the initial lipase solution before immobilization, the supernatant after immobilization, and the PBS washing solution were estimated by the Bradford method. The loading of immobilized lipase was calculated [48].

### 3.7. Characterization

X-ray diffraction (XRD) was performed using a Rigaku Smart Lab diffractometer (Cu-Kα radiation). Transmission electron microscopy (TEM) images were obtained on a Jem-2100F (Japan) instrument. Scanning electron microscopy (SEM) images and energy-dispersive spectroscopy (EDS) maps were recorded with a Hitachi S4800 (Tokyo, Japan). Fourier-transform infrared (FTIR) spectra were tested on a Nicolet iS50 FT-IR spectrometer (Thermo Fisher Scientific, Waltham, MA, USA) in the wavenumber range of 400–4000 cm^−1^ using the KBr pelleting method. Thermogravimetric analysis (TGA) was performed on a Shimadzu DTG-60H simultaneous DTA-TG apparatus, under a nitrogen atmosphere from 30 to 800 °C. The nitrogen adsorption–desorption isotherms were analyzed by a Micromeritics ASAP 2460 analyzer. The magnetism of the samples was measured with a vibrating sample magnetometer (VSM, Quantum Design) at room temperature.

### 3.8. Lipase Activity Test

A certain amount of immobilized lipase was added to PBS (0.025 M) containing 3.4% (*w*/*v*) triacetin, placed in a constant-temperature water bath shaker, 150 rpm, and reacted for 10 min. After the reaction was completed, the immobilized enzyme was collected and titrated with NaOH to calculate the immobilized enzyme activity. In general, one unit of enzyme activity is considered to be the amount of enzyme required to generate 1 μmol of acetic acid per minute [37].

### 3.9. Assays of the Catalytic Performance of the Immobilized Lipase

First, we formulated a triacetin solution with a pH range of 6.0–8.5 and screened the optimal reaction pH at 45 °C. Under the obtained optimal reaction pH, the optimum reaction temperature was selected from within the temperature range of 35–55 °C. Second, the thermal stability of the immobilized enzyme was tested. Under the optimal conditions, a triacetin solution of the sample was incubated on a constant-temperature shaker for 0–6 h, and the enzyme activity was measured and recorded every hour. Then, we evaluated the anti-denaturant properties of the immobilized enzymes. Immobilized enzymes were soaked in urea solution with a concentration of 0–6 M for 2 h and then washed with PBS (pH 7.0). Then, the enzyme activity was measured under the optimal conditions. Finally, we examined the reusability of the immobilized lipase. Under the optimal conditions, we carried out ten recycling experiments using the immobilized lipase.

### 3.10. Measurement of Kinetic Parameters

A certain amount of immobilized lipase was added to the triacetin solution with a substrate concentration of 9–30 mg/mL, and the reaction was carried out at the optimal temperature and pH for 9 min. The initial reaction rate was calculated by titration. The Michaelis constant (*K*_m_) and the maximum reaction rate (*V*_*max*_) were calculated according to the double-reciprocal plot.

## 4. Conclusions

Here, imidazole-based ILs with [PF_6_]^−^ anion-modified Fe_3_O_4_@UiO-66-NH_2_ composites were fabricated and used to immobilize lipase. The prepared supports combined the advantages of magnetic nanoparticles and MOFs and showed a large specific surface area and good magnetic responsiveness. Moreover, the introduction of ionic liquids improved the microenvironment for the immobilized lipase. The results showed that the prepared immobilized PPL exhibited high catalytic activity, excellent stability, and good reusability. The specific activity of PPL-IL-Fe_3_O_4_@UiO-66-NH_2_ was 2.03 U/mg, which was 2.3 times higher than that of soluble PPL. After being reused ten times, the residual activity of PPL-IL-Fe_3_O_4_@UiO-66-NH_2_ was still 74.4%. Kinetic parameters showed that IL-Fe_3_O_4_@UiO-66-NH_2_ had a stronger affinity with the substrate and higher catalytic efficiency. Therefore, IL-Fe_3_O_4_@UiO-66-NH_2_ shows some positive effects for enzyme immobilization. The reaction mechanism between ionic-liquid-modified MOFs and enzymes needs to be further studied to optimize the preparation of immobilized enzymes.

## Figures and Tables

**Table 1:** Results of the immobilized lipase.

Samples	Lipase Content (mg/g)	Expressed Activity (U/mg)	Specific Activity (U/mg)
PPL-Fe_3_O_4_@UiO-66-NH_2_	153.8 ± 5.6	0.30 ± 0.004	1.93 ± 0.03
PPL-IL-Fe_3_O_4_@UiO-66-NH_2_	193.5 ± 3.1	0.39 ± 0.002	2.03 ± 0.01

Immobilization conditions: 30 °C, pH 7.0, 150 rpm, 4 h; the activity of soluble PPL at 45 °C and pH 7.0 is 0.90 U/mg.

**Table 2:** Kinetic parameters of immobilized lipase.

Samples	*K*_m_ (mM)	*V*_*max*_ (μmol·min^−1^·mg^−1^)
PPL-Fe_3_O_4_@UiO-66-NH_2_	75.48 ± 0.03	1.40 ± 0.04
PPL-IL-Fe_3_O_4_@UiO-66-NH_2_	71.74 ± 0.01	1.81 ± 0.01

The *K_m_* and *V*_*max*_ values of soluble PPL were 84.9 mM and 0.78 μmol·min^−1^·mg^−1^, respectively.

## Data Availability

All data are reported in the paper, any specific query may be addressed to liurenmin@lcu.edu.cn (R.L.).
